# Dissociating passage and duration of time experiences through the intensity of ongoing visual change

**DOI:** 10.1038/s41598-022-12063-1

**Published:** 2022-05-17

**Authors:** Mathis Jording, David H. V. Vogel, Shivakumar Viswanathan, Kai Vogeley

**Affiliations:** 1 Cognitive Neuroscience, Institute of Neuroscience and Medicine (INM-3), Forschungszentrum Jülich, Jülich, Germany; 2 Department of Psychiatry, Faculty of Medicine and University Hospital Cologne, University of Cologne, Cologne, Germany

**Keywords:** Human behaviour, Psychiatric disorders

## Abstract

The experience of passage of time is assumed to be a constitutive component of our subjective phenomenal experience and our everyday life that is detached from the estimation of time durations. However, our understanding of the factors contributing to passage of time experience has been mostly restricted to associated emotional and cognitive experiences in temporally extended situations. Here, we tested the influence of low-level visual stimuli on the experience of passage and duration of time in 10–30 s intervals. We introduce a new paradigm in a starfield environment that allows to study the effects of basic visual aspects of a scene (velocity and density of stars in the starfield) and the duration of the situation, both embedded in a color tracking task. Results from two experiments show that velocity and density of stars in the starfield affect passage of time experience independent from duration estimation and the color tracking task: the experienced passage of time is accelerated with higher rates of moment-to-moment changes in the starfield while duration estimations are comparably unaffected. The results strongly suggest differential psychological processes underlying the experience of time passing by and the ability to estimate time durations. Potential mechanisms behind these results and the prospects of experimental approaches towards passage of time experience in psychological and neuroscientific research are discussed.

## Introduction

Time measurements, e.g. as performed by physical clocks, structure our everyday life and enable us to coordinate our actions with others over long distances and long periods, based on the fundamental assumptions that physical time passes by with the same velocity and that it can be reliably measured with clocks. However, everyday experience suggests that this is not how we subjectively experience the passage of time: We refer to time passing by “slowly” when feeling bored or to time “flying” when being engaged in a pleasant activity. In fact, we are aware of this difference and seem to have accepted this decoupling of the experienced subjective passage of time from the ongoing physical time as measured by clocks^1,2^. This is also reflected in changes of the experience of passage of time as described by patients with depressive syndromes^3–6^ and mild cognitive impairments^7^, even when their ability to judge durations is preserved. However, what defines and constitutes the subjective experience of the passage of time as a unique category of time experience remains poorly understood^8–10^.

Prior studies of subjective passage of time experience have focused on the effects of high level emotional or cognitive aspects of activities and situations. Passage of time experience has been associated with the current activity, its hedonic value^11^ and attentional demands^12–14^, the emotional state and the level of anxiety or fear^15–17^, as well as expectations^18,19^, the recall of experiences^20^ and salient contextual factors^8^ regarding a specific interval, and assumptions about time (by manipulating external clocks, for a review see^21^). Nevertheless, the role of low-level stimulus features in modulating the passage of time remains unresolved. To study these features is even more pressing as such features exert a considerable influence on judgements of the *duration* of a time interval as has been already examined in time perception studies^22–26^.

Numerous studies have demonstrated that duration judgments are influenced by the stimuli presented during that interval. For example, for visual stimuli, duration estimations increased with the velocity of moving visual stimuli^27–29^. Accelerating stimuli on the other hand were judged as being shorter in time compared to static stimuli whereas static stimuli were judged as being shorter compared to decelerating stimuli^30,31^. Furthermore, durations were estimated longer for stimuli with larger size, numbers, luminance, and even digit values^32^.

Duration estimations have been used to make indirect inferences about the “speeding up/slowing down” of time-related psychological processes and their neural mechanisms^33–35^. Importantly, however, duration estimations do not require any judgment about the passage of time (which is the focus here). Previous studies suggest that judgments about the passage of time are qualitatively distinct from judgments about the duration of a time interval both for healthy persons^12,36,37^ and for patients with cognitive disorders^3,7^. However, the extensively documented effects of low-level stimulus features on duration judgments raises the question whether these features might also be capable of modulating the subjective passage of time. Furthermore, unlike the sub-second intervals typically investigated for duration judgments, studies of passage of time have typically investigated scenarios involving intervals of several minutes^9,21^. Therefore, in the current study (Experiment 1 in the laboratory, Experiment 2 online), we investigated whether and how the properties of a simple visual stimulus (a field of moving dots) during a supra-second time interval (of 10–30 s) can influence prospective time experience^38^, namely, the subjective experience of the passage of time and its relationship to accompanying effects on judgments about the interval’s duration.

**Figure 1:**
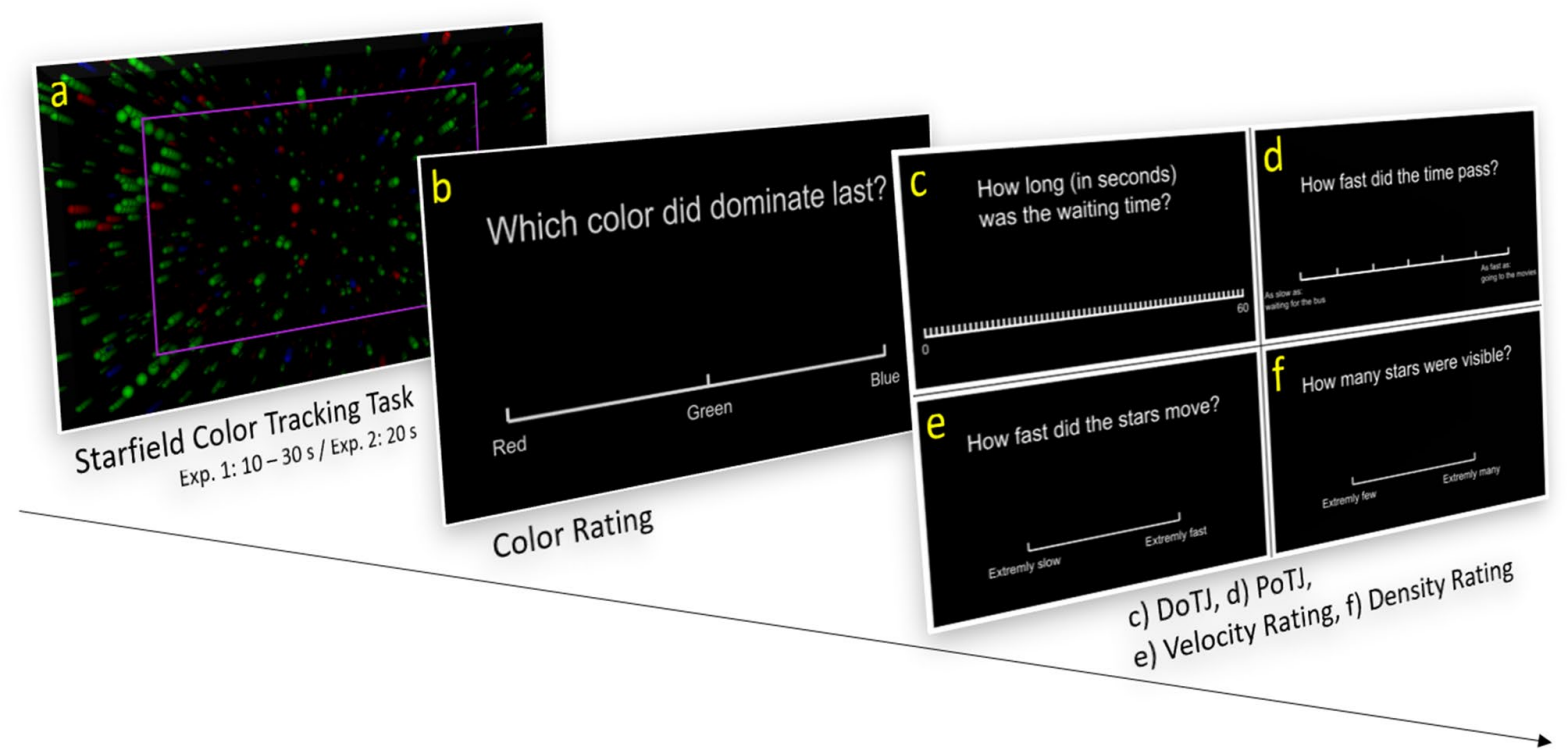
Schematic illustration of the trial course in Exp. 1, including the starfield (**a**), the screen for reporting the dominant color (**b**), and the screens for DoTJs (**c**), PoTJs (**d**), velocity ratings (**e**), and density ratings (**f**). Each trial started with the presentation of the starfield for 10, 20, or 30 s. Immediately afterwards, participants were then asked to report the dominant color, before being either asked to estimate the elapsed time (**c**), to rate the passage of time (**d**), to rate the velocity of the stars (**e**), or to rate the density of the starfield (**f**). The purple rectangle in the starfield screenshot (**a**) was not visible to participant but was added to illustrate the area in which stars would reappear after having reached the periphery of the screen. The course was the same in Exp. 2 but the displays for color reporting and PoTJs differed slightly. For additional information, see supplementary material S1.

In both experiments, the stimulus on each trial was a “starfield” (Fig. 1a)—a randomly distributed collection of filled colored dots (in the following referred to as “stars”) that continuously moved radially from the center of the screen to its periphery over the entire stimulus interval of 10 to 30 s. This created the impression of flying through a cluster of stars without any indicator of when this flight had started or would end. The proportion of star colors in the starfield could also unpredictably change over the stimulus interval. At the end of the stimulus display, participants had to report the dominant color immediately before the stimulus display ended and then judge either the (i) passage of time during the trial, or (ii) the duration of the trial. The demand for color tracking was used to direct participants’ attention to the stimulus and distract them from time-related thoughts, e.g. attention to time.

For the starfield stimulus, the velocity and density are critical determinants of the intensity of visual change. Since the perceived visual change between consecutive frames might be larger for higher velocity and higher density, we reasoned that the moment-to-moment differences between the immediate present and the immediate past might also be perceived as being more salient for starfields of higher velocity and higher density^8^. This potential linkage between visual change and time experience suggests that stimulus velocity and density might exert an influence on the experienced passage of time, irrespective of the physical duration of stimuli. By contrast, the actual duration of the interval might be expected to be the dominant factor that influences duration judgments, which might additionally be modulated by velocity and/or density.

To test these predictions employing the starfield color tracking paradigm, we systematically varied the velocity and density of the stars in the starfield and the trial duration and analyzed their effects on passage of time judgments (PoTJs) and duration of time judgments (DoTJs). We hypothesized that velocity, density, and duration could have dissociable consequences of for DoTJs and PoTJs.

## Experiment 1

### Exp. 1—Methods

#### Participants

20 participants (Age: 18–54, M = 39.3, SD = 10.18; 8 identifying as female, 12 as male) were recruited for the experiment and received monetary compensation (10€ per hour). Inclusion criteria were an age between 18 and 55, exclusion criteria were any record of psychiatric or neurological illnesses, any current psychoactive medication or color blindness. All participants gave their written informed consent before participation in the study. The study was approved by the ethics committee of the Medical Faculty of the University of Cologne, Germany, and adhered to the Declaration of Helsinki. All experiments were conducted in accordance with relevant guidelines and regulations. Note, since neither effect sizes nor intra- or interindividual variance of prospective PoTJs in short time intervals could be estimated from previous studies, the sample size was not based on a-priori power calculation. Instead, we used this study to generate hypotheses in an abductive manner and conducted a second experiment (see below) to test the replicability of central results.

#### Stimuli

The stimuli in our study were characterized by a black background and colored dots (referred to as “stars”) that appear in the center of the screen before moving steadily to the periphery of the screen in a radial fashion and on a straight trajectory. This elicited the impression of moving through a starfield in outer space or a movement in relation to space.

These stimuli were generated using python 3.7.3 (Python Software Foundation, https://www.python.org/) using the PsychoPy^39^ and PyGame^40^ packages, and presented on a 27 inch screen (Asus Swift PG279Q, resolution: 2560 × 1440 pixels).

On each trial, the position and trajectory of all stars were defined in a 3-dimensional space. Each star was initialized at a (uniform) random location within a source rectangle (X =  ± 422, Y =  ± 238, Z = 64), and was assigned one of three colors (red: 255, 0, 0; green: 0, 255, 0; blue: 0, 0, 255) based on a color ratio specified for the entire stimulus (see below). Thus, depending on the specified color ratio, the color composition of the entire starfield was predominated by one of the colors.

Each star then moved on a straight line along the Z-axis until the rectangle Z =  + 128 was reached. When a star reached this target rectangle, it was removed from the display and replaced with a new star at a randomly chosen location within the source rectangle and with a randomly chosen color based on the specified color ratio for the entire stimulus. On each trial, all stars moved in a radial fashion with the same speed. The impression of a dynamic color change was reached through changes in the specified color ratio of the entire stimulus. Subsequently, the whole stimulus would then gradually change the color composition with each star disappearing in the ‘front’ (periphery) and reappearing in the ‘back’ (center) with a new color, chosen based on the new color ratio.

The 3-dimensional motion of the stars were viewed after a perspective projection to a 2D plane at Z = 128 parallel to the source rectangle, with a viewing direction towards the source rectangle. Following this projection, the source rectangle for all the stars had the dimensions (w = 33.19°, h = 18.30° visual angle) at the center of the screen. The prospective projection was also applied to the size of the stars and the brightness.

With the perspective projection, each star was small (diameter = 0.02° visual angle), dimly light, and barely visible when they appeared, and then moved radially along a straight line to the periphery of the screen. On their way, the stars increased in size (max diameter = 0.38° visual angle) and brightness. This approach was chosen to support the participants immersion in a three-dimensional environment and thus to strengthen the effects of the visual stimulation.

Building on this general framework, different variations of the starfield stimuli could be created and displayed. Each particular trial was defined by four parameters: DURATION on three levels (10, 20, 30 s); VELOCITY on two levels (z-increase: 1/s [slow], 4/s [fast]; resulting in an average change in visual angle of 1.08°/s and 3.90°/s), DENSITY on two levels (750 stars [low], 3000 stars [high]), and DIFFICULTY on two levels based on color ratio (4/3 + 3, 6/2 + 2, where the color ratio specifies the proportion of one color (numerator) relative to the two other colors (denominator).

#### Paradigm

Each trial commenced with the display of the starfield stimulus (see Fig. 1a). The end of the stimulus was always marked by a screen displaying the question “Which color dominated in the end?” and the color words “red”, “green”, “blue” (Fig. 1b). Participants clicked on one of these three words to report the most dominant star color at the end of the stimulus. The color rating was then followed by one of four possible questions. These questions were known to participants before the start of the experiment, although participants did not know which particular question would be displayed at the end of a given trial. Nonetheless, participants could certainly expect a time experience related question and therefore DoTJs and PoTJs are considered prospective time judgments^23^.

Two questions were crucial to our study.

1. (‘DoTJ’): “How long was the waiting period?” (scale ranging from 1 to 60 s); (Fig. 1c)
2. (‘PoTJ’): “How fast did the time pass?” (7-point Scale, with extrema labeled with examples for slow and fast passing time from the participants personal everyday life. These examples were based on a pre-experimental interview (see “Procedure” section) and served as autobiographical anchors to give participants additional orientation and to standardize the scales range (Fig. 1d).

The following two questions were included for manipulation checks.

3. (‘velocity’): “How many stars did you see?” (scale ranging from 0 = “extremely few” to 100 = “extremely many”); (Fig. 1e)
4. (‘density’): “How fast did the stars move?” (scale ranging from 0 = “extremely slow” to 100 = “extremely fast”). (Fig. 1f).

Afterwards, the next trial automatically started. The experiment was conducted in two blocks of 15–20 min each. Within a block, each of the 24 unique stimulus combination (3 DURATION levels * 2 VELOCITY levels * 2 DENSITY levels * 2 DIFFICULTY levels) was presented twice in randomized order, once with DoTJ, once with PoTJ. In addition, each block contained 4 manipulation check trials, with all 8 possible combinations of the 2 velocity levels, 2 density levels and 2 manipulation check questions randomly distributed over the whole experiment (duration and difficulty levels were randomly chosen for each manipulation check trial). In total, each block contained 52 trials (24 DoTJ, 24 PoTJ, 4 manipulation check ratings).

#### Procedure

Before the start of the experiment, participants were informed in verbal and written format about the study details and gave their written consent.

After the participant information but before participants had received any more detailed information or instructions about the experiment and the task, a short pre-experimental interview was conducted. In this interview (for a detailed description see supplementary material S2) participants were asked for everyday life examples for fast and slow passing time. These examples, reduced to 1 or 2 keywords would later be used as anchors for PoTJs, individually denoting the minimum and maximum of the PoTJ scales in the computer experiment.

*Experiment:* After the pre-experimental interview, participants were instructed that they would watch different videos showing stars in different colors and that it would be their task to monitor the colors throughout the stimulus and report the lastly predominant color. Since the ratio between the different colors could change at any given point in time, they would have to stay alert and attentive.

Before the start of the experiment, participants were given four practice trials to familiarize themselves with the general trial structure and with the questions (‘color’ + ‘velocity’, ‘color’ + ‘density’, ‘color’ + ‘DoTJ’, ‘color’ + ‘PoTJ). Between the two blocks of the experiment, participants were given the opportunity to take a short break. After the experiment a short post experimental inquiry and questionnaires followed (for a detailed description see supplementary material S3).

#### Data preprocessing

DoTJ raw values were transformed into proportional duration estimates for the analysis by taking the logarithm of the estimated durations divided by the true duration of stimuli (proportional duration estimates = log(Duration_Estimate_/Duration_Real_)). This ensured comparability of DoTJs between different stimulus durations by taking into account the scalar properties of duration estimations^39^. Proportional duration estimates > 0 can be interpreted as overestimations of interval durations while values < 0 mean an underestimation of interval durations. Correspondingly, positive signed coefficients of individual factors would mean that duration estimations are increased while negative coefficients indicate a decrease in duration estimations. Since no equivalent monotonous relationship was expected between the experience of passage of time and the elapsed duration^12,36,37^, PoTJs were not transformed. Subject-wise visual inspection of DoTJs, PoTJs, and ratings in the color detection task suggested that all participants had followed the instructions. No subjects or individual trials had to be excluded from analysis.

#### Statistical analysis

Data were statistically analyzed with R^40^ and RStudio^41^. As recommended for repeated measures designs^42^ we fitted mixed effects models for the analysis of DoTJs and PoTJs, containing random intercept for participants. We treated DoTJs as continuously scaled data and fitted linear mixed effects models with the lmer() function from the lme4 package^43^. PoTJs were treated as ordinal data to which we fitted cumulative link mixed effects models for ordinal regressions with the clmm() function from the ordinal package^44^. Likelihood ratio tests were used to assess the influence of individual predictors by comparing the fit of differently saturated models to a given data set while penalizing the models’ complexity with the anova() function. All statistical tests were performed against an alpha level of p < 0.05. We report the adjusted intraclass correlation coefficient (‘ICC’; performance package^45^) as the ratio of participant wise influences (random intercepts) in the overall variance.

As a manipulation and validity check, we analyzed the effects of star velocity (VELOCITY) on velocity ratings and starfield density (DENSITY) on density ratings. Furthermore, we tested the effect of task difficulty (DIFFICULTY), as well as the additional influences of DURATION, VELOCITY, and DENSITY on the probability for correct color ratings.

We analyzed the influence of interval duration (DURATION), star velocity (VELOCITY), starfield density (DENSITY) and task difficulty (DIFFICULTY) on DoTJs and PoTJs. In order to assess the influence of each of these factors individually, we added them to mixed effects models which we separately compared to null models not including any fixed effects. We then tested for DoTJs and PoTJs whether the stimulus duration or the low-level visual features velocity and density would explain more variance in the participants responses. This was achieved by comparing the fits of the respective models based on which one resulted in a lower (i.e. better fit) AIC (‘Akaike information criterion’^46^). Lastly, we combined all factors for which our first analysis had suggested significant individual effects into full models in order to assess potential interactions between the individual factors. Test statistics for individual coefficients (e.g. p-values) were obtained via the lmerTest() package^47^, using the Satterthwaite’s degree of freedom approximation.

In the following, we report differences in model fit associated with the individual factors as well as the statistics of the corresponding coefficients.

### Exp. 1—Results

For perceptual validity checks and effects of participant variable please refer to the supplementary material S4 and S5.

#### Modulators of color tracking task

DIFFICULTY had a significant effect on the probability of correct color ratings (X^2^(1) = 59.05, p < 0.001), with a probability for a correct rating of 0.61 for easy and 0.44 for difficult color compositions according to the model. Adding DURATION (X^2^(1) = 16.60, p < 0.001) or VELOCITY (X^2^(1) = 14.61, p < 0.001) but not DENSITY (X^2^(1) = 2.24, p = 0.134) further increased the fit significantly. Accordingly, the probability for correct color ratings increased with faster stars (M = 0.34, SE = 0.09) but decreased for each second of stimulus duration (M = − 0.02, SE = 0.01) whereas density did not change the correctness of color ratings.

#### Modulators of duration of time judgments

**Figure 2:**
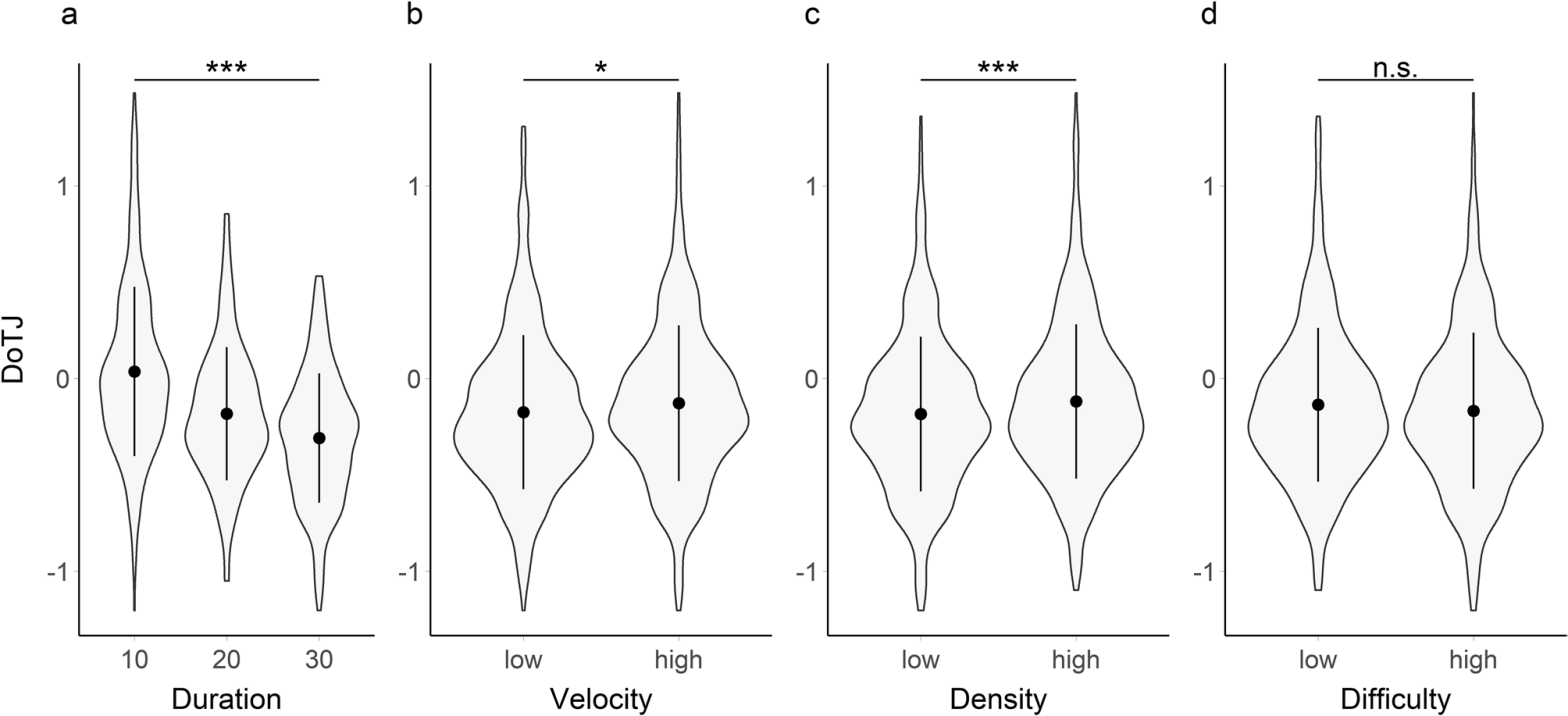
Violin plots of proportional deviation estimates (log(estimated duration/duration)) of DoTJs for the three different stimulus durations (10, 20, and 30 s; **a**), and two different level of velocity (**b**), density (**c**), and difficulty (**d**) in Exp. 1. Proportional duration estimates > 0 can be interpreted as overestimations of interval durations while values < 0 mean an underestimation of interval durations. Central dots indicate the means over all subjects, central vertical lines indicate the sd. Asterisks indicate significant differences between factor levels (* < 0.05; ** < 0.01, *** < 0.001, n.s.: not significant).

For the null model of DoTJs (Fig. 2) not including any other factors than the random intercept for subjects, the ICC suggested that 49.5% of all variance could be explained by consistent differences in duration estimations between participants.

A model that included the stimulus DURATION (Fig. 2a) improved significantly the model fit compared to the null model (X^2^(1) = 261.30, p < 0.001). The corresponding coefficient for DURATION (M = − 0.02, SE = 0.00) implies that underestimation increased with the duration of the intervals (average absolute interval estimations of 10.21 s for the 10 s interval, 17.18 s for the 20 s interval, and 21.68 s for the 30 s interval).

VELOCITY (Fig. 2b) also improved significantly the model fit (X^2^(1) = 6.28, p = 0.012), with the corresponding coefficient (M = 0.05, SE = 0.02) suggesting an increase of the estimated duration by a factor of 1.05 for faster stars.

Similarly, DENSITY (Fig. 2c) improved significantly the model fit (X^2^(1) = 12.23, p < 0.001), with the coefficient for DENSITY (M = 0.07, SE = 0.02) suggesting increased duration estimations by a factor of 1.07 for more dense starfields.

Conversely, DIFFICULTY (Fig. 2d) did not improve the model fit significantly (X^2^(1) = 2.80, p = 0.094).

We then assessed whether the stimulus duration or the combination of the stars velocity and density would have a stronger effect on duration estimations. Here AICs and BICs suggested that the DURATION model (AIC = 166.42) fitted the data better than the VELOCITY + DENSITY model (AIC = 411.13).

Lastly, we assessed potential interactions between the factors for which significant individual effects on duration estimations had been found, i.e. DURATION, VELOCITY, and DENSITY (see above). For this purpose, we fitted a full model (Table 1), including terms for the three factors as well as all interactions between the factors. In accordance with the results from the individual analyses, DoTJs decreased significantly with DURATION (M = − 0.01, SE = 0.00, t(933) = − 6.95, p < 0.001) but increased significantly with VELOCITY (M = 0.13, SE = 0.06, t(933) = 2.10, p = 0.036) and DENSITY (M = 0.19, SE = 0.06, t(933) = 3.17, p = 0.002). Additionally, a significant interaction of DURATION * DENSITY (M = − 0.01, SE = 0.00, t(933) = − 2.09, p = 0.037) suggested that the effect of increased duration estimations was mitigated for longer compared to shorter intervals.

**Table 1:** Summary of the linear mixed effects model for DoTJs (proportional duration estimates), including predictors (as fixed effects) for Duration, Velocity, Density, and all potential interactions.

Predictors	DoTJ (proportional duration estimates)		
	Estimates	CI	p
(Intercept)	0.06	− 0.09 to 0.21	0.428
Duration	− 0.01	− 0.02 to − 0.01	** < 0.001**
Velocity [high]	0.13	0.01–0.24	**0.036**
Density [high]	0.19	0.07–0.31	**0.002**
Duration * velocity [high]	− 0.00	− 0.01 to 0.00	0.206
Duration * density [high]	− 0.01	− 0.01 to − 0.00	**0.037**
Velocity [high] * density [high]	− 0.10	− 0.27 to 0.06	0.224
(Duration * velocity [high]) * density [high]	0.00	− 0.00 to 0.01	0.280
**Random effects**			
σ^2^	0.06		
τ_00 subject_	0.08		
ICC	0.57		
N_subject_	20		
Observations	960		
Marginal R^2^/conditional R^2^	0.132/0.628		

Estimates (in s) and their confidence intervals as well as p-values of the individual predictors can be found in the first part of the table. The intercept refers to a (fictional) condition combination of Duration = 0, Velocity = low, Density = low. The estimates of the other predictors can be interpreted as the expected change in DoTJs in case of a corresponding change in the condition. To account for the repeated measures design, participant wise differences were modeled as random intercept for participants. The second part of the table informs about the random effects (mean variance, ‘σ2’; between subject variance, ‘τ00 subject’, intraclass correlation ICC) as well as some general characteristics of the model (N, Observations, R^2^).

Significant values are given in bold.

#### Modulators of passage of time judgments

**Figure 3:**
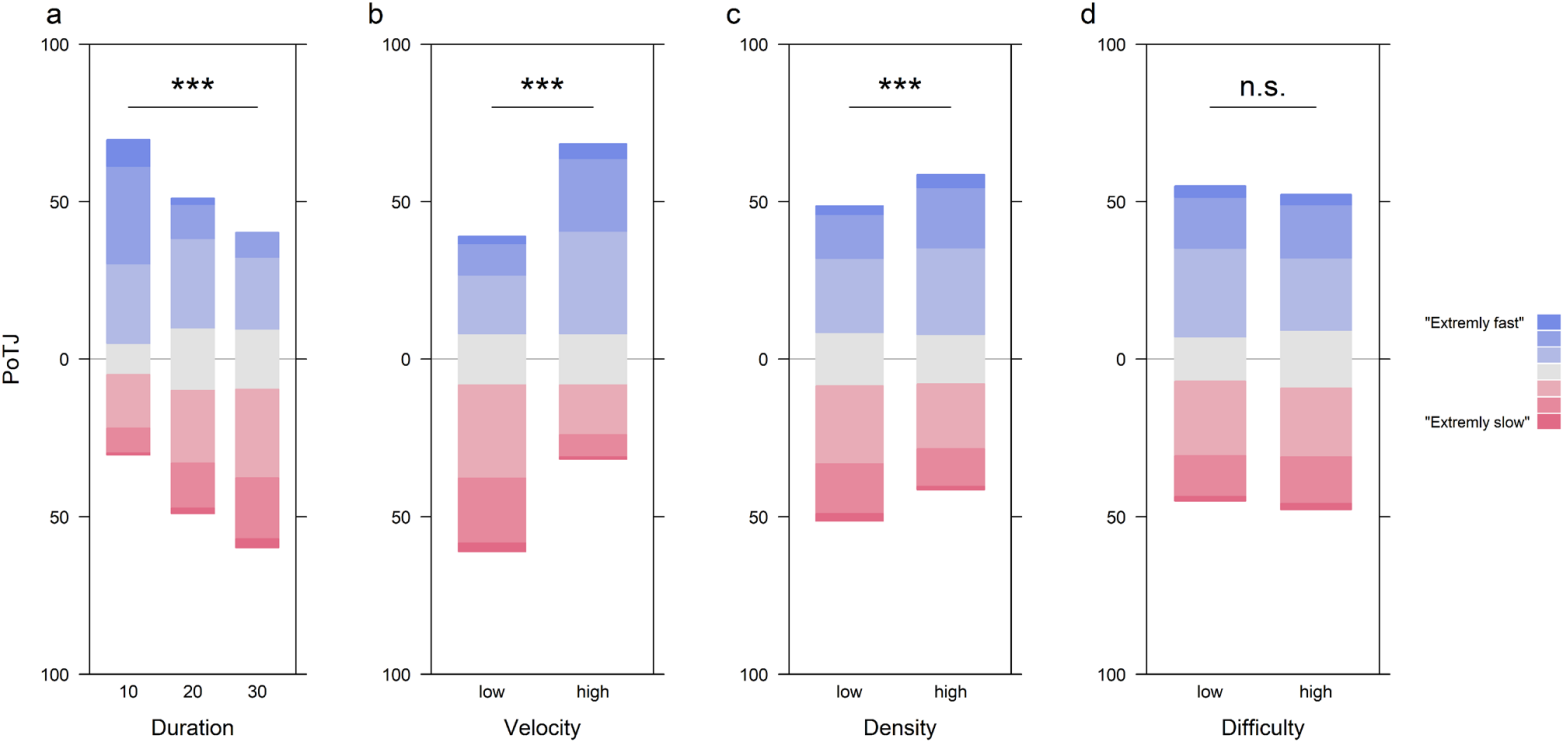
Diverging bar charts of PoTJs for the three different stimulus durations (10, 20, and 30 s; **a**) and two different levels of velocity (**b**), density (**c**), and difficulty (**d**). Each bar represents 100% of all responses within the respective condition with colors (red to blue) illustrating the distribution of responses over the 7 different levels of the PoTJ scale from “extremely slow” to “extremely fast”. In order to enhance comparability between factor levels, bars are aligned to the 4th level (center) of the PoTJ scale. Asterisks indicate significant differences between factor levels (* < 0.05; ** < 0.01, *** < 0.001, n.s.: not significant).

For PoTJs (Fig. 3), the ICC suggested that 23.6% of the variance could be attributed to systematic differences between participants.

Here, too, the inclusion of DURATION (Fig. 3a) increased model fit significantly (X^2^(1) = 148.45, p < 0.001). The coefficients (M = − 0.09, SE = 0.01) suggest that the probability for a higher PoTJ level decreased on average by a factor of 0.91 for each second of stimulus duration.

VELOCITY (Fig. 3b), too, improved significantly the model fit (X^2^(1) = 143.97, p < 0.001), with the corresponding coefficient (M = 1.45, SE = 0.12) implying an increase in the probability for a higher PoTJ level by a factor of 4.28 for higher velocities.

Similarly, including DENSITY (Fig. 3c) improved the model fit significantly (X^2^(1) = 16.83, p < 0.001), with higher density increasing the probability for a higher PoTJ level by a factor of 1.61, according to the coefficient (M = 0.48, SE = 0.12).

As for DoTJs, DIFFICULTY (Fig. 3d) did not significantly improve the fits for PoTJs models (X^2^(1) = 1.13, p = 0.287).

When comparing the influence of stimulus duration to the combined influence of velocity and density, AICs suggested that the VELOCITY + DENSITY model (AIC = 2980.56) fitted the data better than the DURATION model (AIC = 2997.55).

To assess potential interactions between the different factors we again fitted a full model including terms for DURATION, VELOCITY, DENSITY, and their interactions (Table 2). Here, however, only the term for DURATION improved the model fit significantly (M = − 0.12, SE = 0.02, z = − 7.72, p < 0.001).

**Table 2:** Summary of the mixed effects model for PoTJs, including predictors (as fixed effects) for duration, velocity, density, and all potential interactions.

Predictors	PoTJ		
	Estimates	CI	p
1|2	0.00	0.00–0.00	< 0.001
2|3	0.02	0.01–0.05	< 0.001
3|4	0.13	0.05–0.31	< 0.001
4|5	0.36	0.15–0.86	0.021
5|6	2.20	0.93–5.21	0.074
6|7	27.91	11.00–70.83	< 0.001
Duration	0.89	0.86–0.91	< 0.001
Velocity [high]	2.25	0.92–5.49	0.074
Density [high]	1.44	0.59–3.51	0.427
Duration * velocity [high]	1.04	1.00–1.08	0.055
Duration * density [high]	1.01	0.97–1.05	0.571
Velocity [high] * density [high]	1.89	0.54–6.61	0.319
(Duration * velocity [high]) * density [high]	0.97	0.92–1.03	0.304
**Random effects**			
σ^2^	3.29		
τ_00 subject_	1.62		
ICC	0.33		
N_subject_	20		
Observations	960		
Marginal R^2^/conditional R^2^	0.228/0.482		

Estimates (in s) and their confidence intervals as well as p-values of the individual predictors can be found in the first part of the table. The intercept refers to a (fictional) condition combination of Duration = 0, Velocity = low, Density = low. The estimates of the other predictors can be interpreted as the expected change in DoTJs in case of a corresponding change in the condition. To account for the repeated measures design, participant wise differences were modeled as random intercept for participants. The second part of the table informs about the random effects (mean variance, ‘σ2’; between subject variance, ‘τ00 subject’, intraclass correlation ICC) as well as some general characteristics of the model (N, Observations, R^2^).

### Exp. 1—Discussion

Consistent with the experimental manipulation, duration judgments (DOTJs) were influenced by the corresponding objective stimulus durations but with the longer intervals being more underestimated than the shorter intervals (Fig. 2a). Similarly, judgments of the passage of time (PoTJs) were also modulated by the objective stimulus duration, with the passage of time being judged to be slower for longer durations (Fig. 3a). Importantly, the low-level features of the visual stimulus (velocity and density) modulated both the DOTJs and POTJs. Intervals with a faster and denser starfields had DOTJs with a marginally smaller deviation from the objective durations (Fig. 2b,c). However, for POTJs, faster and denser starfields were judged to have a faster passage of time (Fig. 3b,c).

Based on their explanatory power, stimulus duration and the low-level visual features (i.e., velocity and density) differed in their relative influence on DOTJs and POTJs. Stimulus duration had a more decisive influence on duration estimations than the low-level visual features. This was reversed for POTJs, which were more strongly influenced by the visual features as compared to stimulus duration. Together these findings suggest that the low-level stimulus features that are known to effect duration judgments can also have a substantial influence on judgments of the passage of time.

Furthermore, the differences in the relative explanatory power of duration vs. low-level visual features in DoTJs and PoTJs suggest that duration estimation and passage experience might be based on different mechanisms. The effect of stimulus duration on DOTJs and POTJs points towards another phenomenological distinction between the two types of time experience. It is noteworthy that when estimating duration, longer intervals (e.g., 30 s) were underestimated to a greater extent than a short interval (e.g., 10 s). This underestimation pattern would seem to suggest that the longer intervals were implicitly experienced as running relatively *faster*. This stands in contrast to the explicit passage of time judgments where longer intervals were judged to have a *slower* passage of time. This relationship would suggest that DOTJs and POTJs were not mere derivatives of each other. Note, however that in this experiment participants performed prospective time judgments, which are presumably based on different cognitive processes compared to everyday life situations where the passage is usually judged retrospectively^48^. Furthermore, since participants were not informed before a trial whether they had to provide either a DoTJ or PoTJ, both types of judgments could have potentially interfered with each other.

To further corroborate the notion of the qualitative distinction between DoTJs and PoTJs and also to confirm the effects of visual stimulus features especially on PoTJs, we conducted a second experiment with some adjustments to the general approach. Data from Exp. 1 did not give any reason to suspect a significant influence of task difficulty on either DoTJs or PoTJs. However, significant differences in color detection accuracy between trials of different durations and different velocities could suggest a confounding of task difficulty and interval duration and star velocity. This may point to a potential limitation of Exp. 1, namely the fact that we did not systematically control the time points of color changes in the starfield. Since the starfield changed its color composition gradually with every star that reached the front and reappeared with a new color in the back, the speed of the whole process depended on the velocity of the stars. Thus, color composition changes were more abrupt for faster stars and especially color changes immediately before the end of a trial would be easier to detect in case of faster moving stars.

## Experiment 2

We decided to drop the different intervals and difficulty levels, instead focusing on the effects of velocity and density on DoTJs and PoTJs. In addition, we now controlled closely the number and time points of color changes in each trial, ensuring a balance between the conditions and preventing the occurrence of color changes in the last 1.5 s of a trial. Most importantly, every participant was either asked to respond to DoTJs or to PoTJs (but not both during the experiment) in order to separate both tasks between subjects and prevent potential confusions of the two. We also decided to investigate the effects of velocity and density within and between subjects. These changes in design required a higher number of participants compared to Exp. 1, because of which Exp. 2 was conducted online. Furthermore, the design changes meant that effect sizes were not readily transferable between experiments and thus the minimum sample size for each comparison was set to 40 participants (For a more detailed analysis of sample size and resulting power see below).

### Exp. 2—Methods

#### Participants

179 participants (Age: 18–55, M = 28.68, SD = 7.89; 59 identifying as female, 119 as male, 1 as diverse) were included for the analysis of this study. Due to data-quality considerations, an additional 85 participants had to be excluded due to different reasons (for more details see data-quality assessment in supplementary material S6). Participants were invited via the platform prolific.co with the same inclusion and exclusion criteria applying as in Exp. 1 (no record of psychiatric or neurological illnesses, age between 18 and 55, no current psychoactive medication, no color blindness). All participants gave their informed consent before participation in the study and were monetary compensated with £7.50 per hour. The study was approved by the ethics committee of the Medical Faculty of the University of Cologne, Germany, and adhered to the Declaration of Helsinki. All experiments were conducted in accordance with relevant guidelines and regulations.

#### Stimuli

Exp. 2 again made use of starfield stimuli with the same general structure as the stimuli in Exp. 1. All stimuli were created with a similar algorithm as in Exp. 1 (see Exp. 1/“Stimuli” section; for changes as adjustments to the online setting of Exp. 2, see supplementary material S7).

Exp. 2 did not entail a variation in task difficulty but the color ratio was always 80% for the predominant color vs. 10% for each of the other two colors. Number and onset of changes in color ratios were now fully controlled compared to Exp. 1. Per combination of star velocity and density, 10 videos were created (40 in total) containing different numbers of changes: 1 video containing 3 changes, 3 videos containing 2 changes, 6 videos containing 1 change. These changes were evenly spread over the duration of the trial.

In total, four groups of stimuli were created, one for each combination of velocity (slow vs. fast) and density (low vs. high).

#### Trial design

The design of the experiment itself was very similar to Exp. 1, only that now conditions were varied in a mixed within- and between-subject design (instead of the pure within-subject design of Exp. 1). To this end, participants were split into 8 groups (Table 3). Each of the groups perceived trials that contained either changing velocity (Table 3, row 1) or changing density levels (Table 3, row 2). The other of the two modalities (velocity or density) was kept at a constant low or high level (Table 3, 2nd and 3rd line in each cell). In addition, each group always had to give either DoTJs or PoTJs (condition ‘judgment’), but not both. Thus in summary, a 2 * 2 * 2 design (change modality * intensity level * judgment) resulted. Participants were neither informed about the whole design nor about the specific trial manipulation they were assigned to.

**Table 3:** Condition combinations of the 8 experimental groups, each containing a minimum of 20 participants.

		Judgment	
		DoT (n = 82)	PoT (n = 97)
(Within subject) manipulation	Velocity (n = 80)	n = 40	n = 54
		Group 1 (n = 20): low density	Group 5 (n = 33): low density
		Group 2 (n = 20): high density	Group 6 (n = 21): high density
	Density (n = 80)	n = 42	n = 43
		Group 3 (n = 21): low velocity	Group 7 (n = 23): low velocity
		Group 4 (n = 21): high velocity	Group 8 (n = 20): high velocity

Each group had to provide either DoTJs or PoTJs (columns) and was confronted with changes in one of the two modalities velocity and density (rows), while the other was kept constantly low or high (2nd and 3rd line in each cell).

#### Procedure and task

Task and instructions were highly similar to the procedure in Exp. 1 with the key difference that each participant only received instructions according to their respective judgment condition.

To each participant 20 (10 low + 10 high velocity or 10 low + 10 high density) trials were presented in randomized order but with uniform structure. Each trial started with the presentation of a starfield video, embedded in a page with black background and without any visible border. Unbeknownst to the participants, each video lasted exactly 20 s. After the video, participants were first asked to report the last predominant color before they were then asked either for a DoTJ or PoTJ, according to their assigned judgment condition. DoTJs were entered as a number of seconds in a text box while PoTJs were reported via a slider on a scale ranging from “very slow” to “very fast” (in 100 steps). Afterwards, the next trial started automatically after a short pause caused by loading the stimuli (the duration depended on the participants device and internet connection).

For procedures specific to the online format of this study please refer to the supplementary material S8.

#### Statistical analysis

Data analysis was conducted in accordance with the procedure from the analysis for Exp. 1. The effects of stimulus velocity and density were again tested in likelihood ratio tests of differently saturated models. However, PoTJs were now recorded on a scale (100 steps) that can be considered continuous for the purpose of this analysis and therefore it was also modeled as a linear model. This experiment did not entail a manipulation of stimulus durations and task difficulty. Instead, the split into different experimental groups (see “Trial design” section; Table 3) allowed for an additional investigation of effects between groups. Therefore, we fitted linear models and linear mixed effects models entailing random intercepts to account for between subject variations. All tests for statistical significance were performed against a level of 0.05 (additionally corrected for multiple comparisons in the case of post-hoc tests, see below).

As a first step and in parallel to the analysis of Exp. 1, we tested the effects of variations of VELOCITY and DENSITY on DoTJs and PoTJs within subjects. Since in each group of participants only one factor (VELOCITY or DENSITY) was manipulated and each group was only asked to provide either DoTJs or PoTJs (see Table 3), the effects were analyzed separately. For the analysis of the effect of VELOCITY, the low and high DENSITY groups were pooled (Groups 1 & 2 in DoTJ; 5 & 6 in PoTJ) and for the effect of DENSITY, low and high VELOCITY groups were pooled (Groups 3 & 4 in DoTJ; 7 & 8 in PoTJ). The starting models here already included terms for the effect of the respective between group manipulation in order to isolate the influence of the within-group manipulation. The ICC as a measure of overlap and variances between participants was computed separately for each group to exclude influences due to systematic differences between groups. In addition, models included a random intercept to account for the repeated measures design and subject specific effects. In order to elucidate interaction effects and to assess effects for each individual group, we conducted Tukey post-hoc tests for the comparison between individual factor levels (correcting for multiple comparisons) with the emmeans() function from the emmeans package^49^.

Afterwards, we also analyzed the effects of VELOCITY and DENSITY on DoTJs and PoTJs between subjects. Here, for DoTJs and PoTJs we computed the average for each participant over all trials. For the analysis of DoTJs (Fig. 5a,b) we compared groups with low VELOCITY (Group 3) and high VELOCITY (Group 4) and low DENSITY (Group 1) and high DENSITY (Group 2). Accordingly, for the analysis of PoTJs (Fig. 5c,d) we compared groups with low VELOCITY (Group 7) and high VELOCITY (Group 8) and low DENSITY (Group 5) and high DENSITY (Group 6).

#### Power analysis

We analyzed the power in Exp. 2 based on the sizes of effects found in Exp. 1. Note, that this can only be an approximation since several differences exist in the experimental design between the two experiments and that calculations of observed power can be imprecise when based on results from single studies^50^.

For the power analysis, mixed effects models including random intercepts for participants and the factors duration, velocity and density as well as their interactions were fitted to DoTJ and PoTJ data from Exp. 1. The resulting model coefficients were then used as population parameters for the sampling of new sets of trials based on the number of participants and trials in Exp. 2. Four separate power calculation were conducted for the analyses of velocity and density in DoTJs and PoTJs. For each of the four calculations, 10,000 data sets were drawn, each consisting of 800 trials (40 participants with 20 trials each). In each of the four power calculations, it was then tested for each of the 10,000 data sets, whether including the respective factor (e.g. velocity) would improve the model fit significantly either as a main effect or via an interaction with the other factor (e.g. density). The power was then approximated as the ratio of significant results in the total number of repetitions (10,000). This procedure is based on and explained in more detail in DeBruine and Barr^51^. In DoTJs, the power for the analysis of velocity effects was 0.68 and for density 0.93, in PoTJs the power was 1.0 for velocity and 0.99 for density.

### Exp. 2—Results

#### Validity check

Exp. 2 did not contain checks of the effect of speed or density manipulations. The overall accuracy in the color detection task was 0.966. As in Exp.1 we tested the effect of star VELOCITY and DENSITY on the probability for correct color ratings by comparing the model fit of generalized mixed effects models including a random intercept for participants in likelihood ratio tests. VELOCITY (X^2^(1) = 29.72, p < 0.001), but not DENSITY (X^2^(1) = 1.60, p = 0.206) significantly affected the probability for correct color ratings with 0.951 for slow and 0.981 for fast stars (probabilities separated by VELOCITY: low = 0.962; high = 0.970).

#### Modulators of duration of time judgments

**Figure 4:**
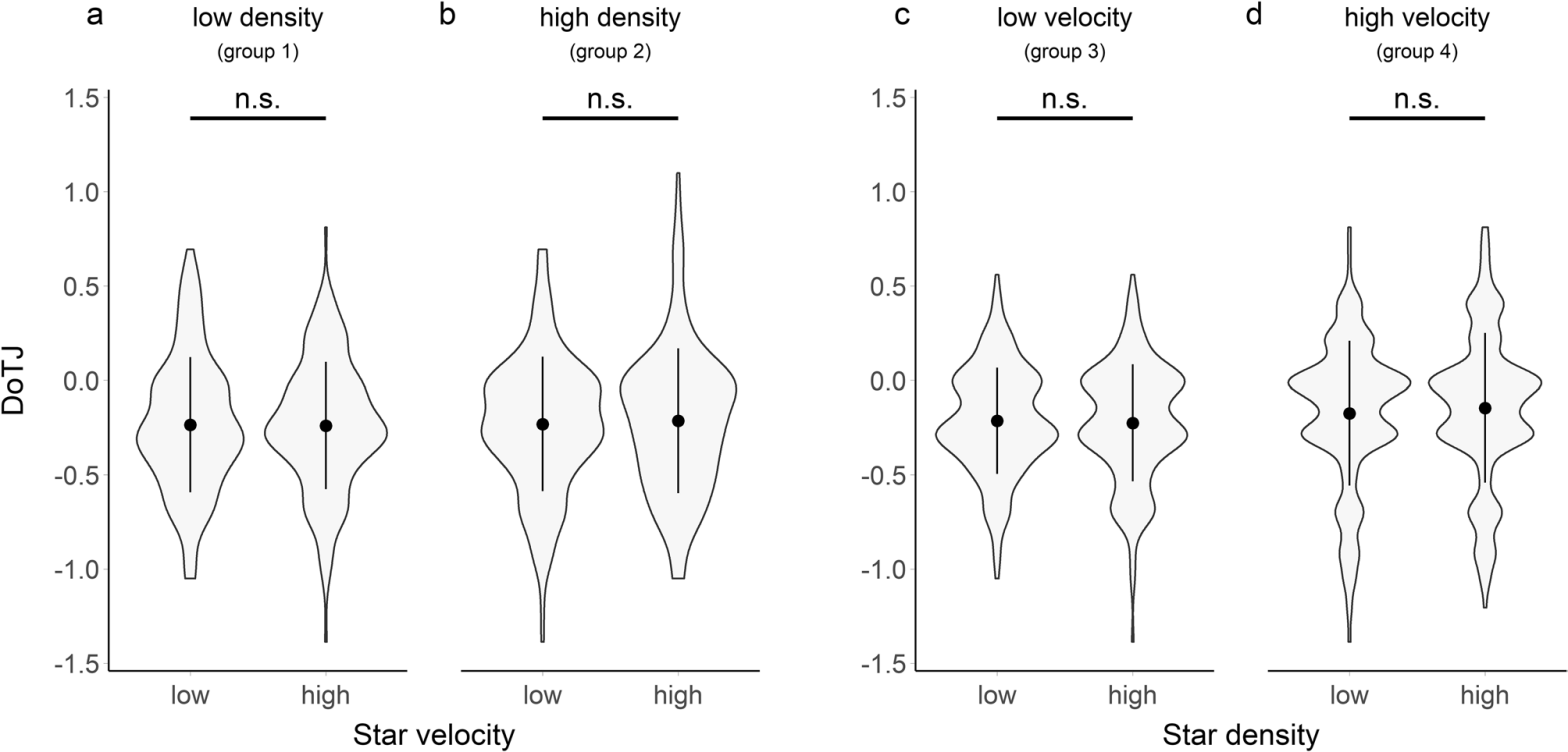
Violin plots of proportional deviation estimates (log(estimated duration/duration)) of DoTJs (data from groups 1–4). In the groups 1 & 2 (**a**,**b**) the velocity of stars was manipulated (within-subject manipulation), while density was kept at a constant level (Group 1: low; Group 2: high). In the groups 3 & 4 (**c**,**d**) the density of stars was manipulated (within-subject manipulation), while velocity was kept at a constant level (Group 3: low; Group 4: high). Proportional duration estimates > 0 can be interpreted as overestimations of interval durations while values < 0 mean an underestimation of interval durations. Central dots indicate the means over all subjects, central vertical lines indicate the sd. Asterisks indicate significant pairwise Tukey post-hoc test (* < 0.05; ** < 0.01, *** < 0.001, n.s.: not significant), corrected for multiple comparisons.

In DoTJs, in groups 1 & 2, experiencing changes in velocity, 57.2% (Group 1; Fig. 4a) and 65.6% (Group 2; Fig. 4b) of all variation was explained by systematic differences between participants. In the groups 3 & 4, experiencing changes in density, this ratio was 44.9% (Group 3; Fig. 4c) and 74.4% (Group 4; Fig. 4d).

##### Within-subject modulators of duration of time judgments

In the comparison of trials of different VELOCITY (groups 1 & 2; Fig. 4a,b), neither including a term for VELOCITY (X^2^(1) = 0.14, p = 0.707) nor the interaction VELOCITY*DENSITY (X^2^(1) = 0.57, p = 0.450) improved model fits significantly. Accordingly, post-hoc pairwise comparisons did not reveal any significant effects of VELOCITY, neither in the low density (M = 0.01, SE = 0.02, t(730.14) = 0.27, p = 0.993) nor the high density group (M = − 0.02, SE = 0.02, t(730.14) = − 0.80, p = 0.855).

Similarly, in the comparison of trials of different DENSITY (groups 3 & 4; Fig. 4c,d), neither including a term for DENSITY (X^2^(1) = 0.01, p = 0.915) nor the interaction DENSITY*VELOCITY (X^2^(1) = 0.34, p = 0.560) led to a significant improvement of model fits. Again, post-hoc pairwise comparisons did not reveal significant effects of density in the low velocity (M = 0.01, SE = 0.02, t(758.11) = 0.34, p = 0.987) or the high velocity group (M = − 0.01, SE = 0.02, t(758.16) = − 0.48, p = 0.962).

##### Between-subject modulators of duration of time

When comparing DoTJs between groups of different VELOCITY levels (group 3 vs 4), model fits were not significantly improved by including the effects of VELOCITY (F(1,40) = 0.60, p = 0.441).

The same was true for the effect of DENSITY (F(1,38) = 0.02, p = 0.892), which also did not improve the fit in the comparison between the groups of different DENSITY levels (group 1 vs. 2).

#### Modulators of passage of time judgments

**Figure 5:**
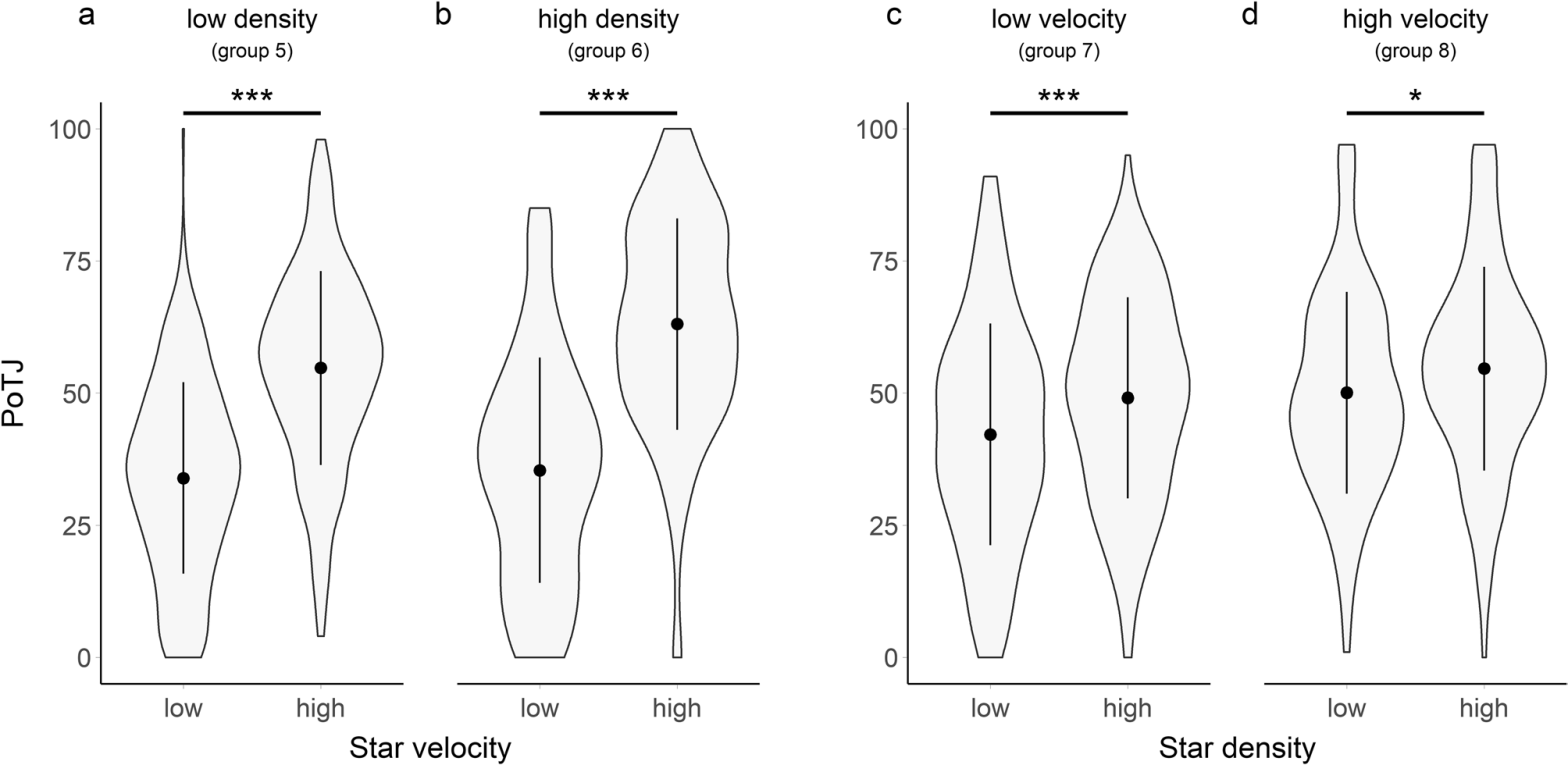
Violin plots of PoTJs (data from groups 5–8). In the groups 5 & 6 (**a**,**b**), the velocity of stars was manipulated (within subject manipulation), while density was kept at a constant level (Group 5: low; Group 6: high). In the groups 7 & 8 (**c**,**d**) the density of stars was manipulated (within subject manipulation), while speed was kept at a constant level (Group 7: low; Group 8: high). Central dots indicate the means over all subjects, central vertical lines indicate the sd. Asterisks indicate significant pairwise Tukey post-hoc test (* < 0.05; ** < 0.01, *** < 0.001; n.s.: not significant), corrected for multiple comparisons.

In PoTJs, ICCs suggest that in groups 5 & 6, experiencing changes in velocity, 14.6% (Group 5; Fig. 5a) and 24.6% (Group 6; Fig. 5c) of all variation was explained by systematic differences between participants. In the groups 7 & 8, experiencing changes in density, this ratio was 52.3% (Group 7; Fig. 5c) and 50.6% (Group 8; Fig. 5d).

##### Within-subject modulators of passage of time judgments

In the comparison between trials of different VELOCITY (groups 5 & 6; Fig. 5a,b), the term VELOCITY (X^2^(1) = 425.17, p < 0.001) as well as the term for the interaction VELOCITY*DENSITY (X^2^(1) = 10.21, p = 0.001) improved model fits significantly. The coefficients in the resulting model suggested that PoTJs were decreased for slower compared to faster trials (M = 20.86, SE = 1.30), with the effect decreasing in interaction with low density (M = 6.64, SE = 2.07).

In post-hoc pairwise comparisons (Tukey corrected for multiple comparisons), lower velocity showed significant effects in both the low density group (M = − 20.86, SE = 1.30, t(981.61) = − 16.06, p < 0.001) and the high density group (M = − 27.50, SE = 1.62, t(981.43) = − 16.99, p < 0.001).

In the comparison between trials of different DENSITY (groups 7 & 8; Fig. 5c,d), including the effect of DENSITY (X^2^(1) = 34.22, p < 0.001) but not the interaction DENSITY*VELOCITY (X^2^(1) = 1.91, p = 0.167) improved model fits significantly. Accordingly, PoTJs were decreased for low density compared to high density trials (M = 1.72, SE = 3.17).

In post-hoc pairwise comparisons (Tukey corrected for multiple comparisons), lower density showed significant effects in both the low velocity (M = − 6.83, SE = 1.30, t(793.10) = − 5.25, p < 0.001) and the high velocity group (M = − 4.21, SE = 1.38, t(793.06) = − 3.05, p = 0.013).

##### Between-subject modulators of passage of time judgments

In the comparison of PoTJs between groups of different VELOCITY levels (group 7 vs 8), including VELOCITY did not significantly improve the model fit (F(1,41) = 2.41, p = 0.128).

Similarly, when comparing PoTJs between groups of different DENSITY levels (group 5 vs. 6), the inclusion of DENSITY did not significantly improve the model fit (F(1,52) = 2.83, p = 0.098).

### Exp. 2—Discussion

In contrast to Exp. 1, in Exp. 2 only velocity and density of the starfield were manipulated while interval duration and task difficulty were kept constant. Each participant only provided DoTJs or PoTJs and experienced changes in only one modality, either velocity or density. The groups of participants experiencing changes in velocity were further split into one low and one high density group and vice versa. This allowed the analysis of effects between as well as within subjects. Contrary to the results from Exp. 1, in Exp. 2 DoTJs did not show any significant influence of either velocity or density, neither between- nor within subjects. However, the effect of increased PoTJs for higher velocity and higher density could be confirmed in the within subject analysis. Furthermore, the PoTJ increasing effect of velocity was further increased in the high density group. The between subject analysis of PoTJs did not reveal significant differences. However, note that the analysis of velocity effects on DoTJs was underpowered (see "Power analysis" section) and thus it is not possible to generally rule out the existence of effects comparable in size to those found in Exp. 1.

In principle, the paradigm also allows to assess the influence of participant specific effects in DoTJs and PoTJs by comparing the ICCs^52^. Since we had no prior hypothesis regarding ICCs we will refrain from speculating about the potential differences between DoTJs and PoTJs suggested by the results for Exp. 1.

The paradigm does require thoroughly controlling all aspects related to the color tracking task as apparent from the low accuracy rates in color ratings in Exp. 1. In Exp. 2, especially the number and time point of color changes were controlled more closely, resulting in higher overall accuracy scores. This was necessary to exclude potential confounds between experimental conditions, prevent frustration in participants and to generally ensure a closer control over the participants experience while doing the task. It has to be noted that in Exp. 2 we again observed a significant difference in accuracy depending on the speed of stars. However, the resulting difference in the accuracy was small (0.95 vs 0.98) and most likely not noticeable for participants.

## General discussion

In this study, we investigated the experience of how fast time passes in time intervals in the range of decades of seconds and whether it would be susceptible to manipulations by low-level stimulus features and/or task characteristics. This study shows for the first time that passage of time experience can be manipulated in the sub-minute range through low-level visual stimuli. Furthermore, using the starfield color tracking paradigm, the experience of passage of time and estimation of the duration of an interval can be experimentally differentiated under laboratory conditions.

Passage of time experience was increased for faster stars and more dense starfields, but was not as much affected by the actual duration of the interval or the task difficulty. This shows that the salience of moment-to-moment differences between individual frames is more directly associated with the experience of passage of time than is the actual duration of the interval. This is in line with the notion that the phenomenal experience of passage of time depends on the most salient internal or external contextual changes^8^ or the relationship between the unfolding of events in the external world (e.g. starfield) and internal (e.g. bodily) processes^53^. Accordingly, trials with high velocity or high density and thus higher rates of moment-to-moment change felt faster in relation to less salient changes or the unchanged speed of internal processes.

Analyzing the effects of the experimental manipulations on DoTJs, we found—as predicted by “Vierodts’ Law”^27,54^—that DoTJs were found to be influenced by the stimulus durations with more pronounced underestimations of longer intervals. Previous studies have also found duration estimations to be susceptible to manipulation by low level visual stimuli^27–32^. However, in our study duration estimations were only affected if participants were trying to assess both types of time experience in parallel in Exp. 1. When participants were instructed to explicitly focus on either passage experience or duration estimation in Exp. 2, duration estimations were not influenced by low level features any more. This suggests that participants might have not been able to give entirely independent reports of the two aspects of time experience when not being informed before the trial which judgment would be required in Exp. 1. This raises the question to which extent earlier results about the effects of visual stimuli on duration estimations^27–29,32^ might be attributable to similar effects. As pointed out, humans are aware of the discrepancy between the passage and duration of time in everyday life on a conceptual level^1,2^. However, the question remains to which extent this insight is reflected when actually reporting on time experience.

Judgments about the “passage of time” are indicators of a category of subjective experience unlike the conceptual notions of “passage of time”, for example, when duration judgments and neuroimaging data are used to draw inferences about the “speeding up/slowing down” of time related psychological or neural processes^33–35^. The qualitative distinction between these experiences has repeatedly been emphasized^12,36,37^. However, empirical investigations of this relationship so far have proven to be complicated. For many existing studies about the relationship of DoTJs and PoTJs it is difficult to assess the validity of the results, as in these studies the two judgments in fact relate to two different situations^12,36,37,55^. Passage of time was reported for a situation which participants experienced up until a cue (either as part of their everyday life or as part of an experimental paradigm) while durations were estimated for an interval presented afterwards. Surprisingly few studies about time perception entailed assessments of duration estimations and passage judgments for the same situations^13,14^. Some additional older studies and/or focusing on topics other than time perception per se are summarized in Thönes et al.^21^. With exception of one^14^, these studies seem to have found concurrent effects in duration estimations and time passage experience. Other studies demonstrated that under certain circumstances, the estimation of durations and experience of passage of time can indeed influence each other. In two studies^11,18^ passage of time reports were altered by manipulating either the prediction of a duration or information about its actual duration. Similarly, presenting participants with unbeknownst manipulated (accelerated or decelerated) physical clocks could affect their experience of passage of time (for a review see^21^).

With regard to the origin and mechanism behind the effects of velocity and density on the experience of passage of time, two potential explanations can be discussed, either (1) direct influences on an internal passage of time state (“bottom-up”) or (2) influences by metacognitive concepts of passage of time experience (“top-down”).

The notion that passage of time experience is directly affected by situational factors (1) assumes that participants have access to and report directly on internal bodily states that constitute their experience of passage of time. Passage of time would thus be experienced similar to sensations or emotions. In the past, different situational factors have been identified to determine the experience of passage of time in longer time ranges than studied here. So far, passage of time experience has been linked mainly to attentional demands of activities^12–14^ or their hedonic value^11^. The experience of “flow” is often described as mechanism behind these effects on the experience of passage of time^56^.

However, we did not find any evidence that task difficulty as a potentially attention mediating factor in the starfield color tracking paradigm had a substantial effect on passage of time experience. Manipulations of the starfield’s velocity and density on the other hand were irrelevant for the attentional demands of the task. Likewise, it seems implausible that the effect of manipulations of visual features on passage of time experience was mediated by an influence on the current emotional state of the participants—another factor that has been reported to affect passage of time experience in the past^15–17^. The most convincing argument for the internal state hypothesis would certainly be finding some kind of signal (bodily or neural) that reliably predicts PoTJs across different contexts.

Another possible explanation for the effects of velocity and density requires the assumption of metacognitive judgments or reconstructions of primary passage of time experience (2). As pointed out by several authors, currently the basis for participants reports of their experience of passage of time is unclear^1,9^. Thus, instead of reports reflecting experiences during the specific situation, they could be inferred based on mental concepts of time experience^9,57,58^. According to this view, the experience of passage of time would not emerge in the situation itself but it would be constructed only afterwards when participants were asked to report on it. It could well be that participants—knowingly or unknowingly—associated movement and density in their imagination with more interesting activities and in turn with faster passing of time and this metacognition superimposed their PoTJs. However, our everyday understanding of time as passing slowly when being bored and fast when being engaged in interesting activities usually refers to situations which are usually in the range of minutes or even hours, these judgments are usually made retrospectively. This is different from and not fully comparable to the laboratory situation in this study where participants were asked to make time judgments for durations ≤ 30 s prospectively. Consequently, PoTJs in this task should not be very prone to superimpositions by cognitive concepts of passage of time.

Especially the association between the velocity of stars and the speed with which time passes could have been further emphasized by the wording of the PoTJ question “How *fast* did time pass?”. The design of this study does not allow to control for the specific effect of the speed metaphor on the judgment. However, the contextual cues entailed in the prompt would not explain the other effect of low-level visual stimuli properties, i.e. density. Nonetheless, it seems worth examining the impact of the wording of PoTJ prompts in future investigations of the effect of low-level visual stimuli on the experience of passage of time.

Since PoTJs as requested in our study in itself already involve higher cognitive processes, ruling out any kind of metacognitive influences on the decision does not seem possible. Therefore, the probability of the metacognitive hypothesis being true can only be assessed by systematically ruling out other mechanisms like e.g. suggested by the internal state hypothesis. Additional experiments, specifically designed to compare and test alternative mechanisms will be necessary to decide which concept of passage of time experience is more adequate.

In conclusion, in this study we were able to show that low-level visual stimuli can affect passage of time experience in short time intervals. Furthermore, with the starfield color tracking task we have introduced a paradigm which allows to experimentally manipulate passage of time experience under controlled laboratory settings and compare it to other aspects of time processing such as duration estimation. Especially the ability to systematically modify the experience of passage of time in a laboratory setting might entail the opportunity for a more thorough search for the (neuro)physiological correlates of the experience of passage of time. This potentially enables investigations into the neural correlates of time experience and their variances in psychiatric conditions with prospects of an incorporating time experience in clinical diagnostics and treatment^4–6^. One question that should be addressed is whether the background of effects in passage of time rely on internal states and/or on metacognitive judgments. This admittedly ambitious endeavor promises to further elucidate the nature of the experience of time passing by.

## Supplementary Information

Supplementary Information.

## Data Availability

Data and analysis code publicly accessible via: https://osf.io/u9pz3/?view_only=b2f5a81f93144c44b99985135d957ef9.
