# Supplementary material for: Dissociating passage and duration of time experiences through the intensity of ongoing visual change
Source: Sci Rep. 2022 May 17;12:8226. doi: 10.1038/s41598-022-12063-1 (PMC9113985; doi:10.1038/s41598-022-12063-1)
Supplement: Supplementary file 1 — Supplementary Information. [file 41598_2022_12063_MOESM1_ESM.docx]

**Supplementary Material**

*Jording et al., “Dissociating passage and duration of time experiences through the intensity of ongoing visual change”*

**S1) Description Figure 1**

For the purpose of illustration, a few changes were applied compared to the original material: To illustrate the movement of stars in the starfield, five consecutive frames of the starfield are shown as an overlay. The screenshots of the starfield itself were taken from Exp. 2 (low velocity / high density condition) since here fewer but larger stars were presented, making it more suitable for a schematic illustration. The screens for the color rating and PoTJ were rebuild with English instead of the original German text. Text and rating scale are also increased in size in relation to the screen size.

**S2) Exp. 1 – Methods – Pre-experimental interview**

In this interview, participants were asked: 1. Whether they experience the passing of time, how they know, that time passes, and whether they had ever experienced time standing still; 2. Whether they experience that the passing of time can change and to give everyday life examples of situations in which the time passes especially fast and situations in which time passes especially slow. Lastly, participants were also asked whether they have the feeling that the time passes differently for them compared to other persons.

The first part of the interview (1.) was conducted to assess the participants’ intuitive attitudes towards time and their experiences of the passing of time. Information will be analyzed qualitatively in the future and served exploratory purposes.

Information from the second part of the interview (2.) about everyday life examples for fast and slow passing time were used in the following experiments as anchors for the passage of time scales for PoTJs. Therefore, the experimenter summarized each example in 1-2 keywords reassuring with the participants that these keywords would clearly identify and reflect the situation. The keywords would later be displayed, individually denoting the minimum and maximum of the PoTJ scales in the computer experiment for every participant.

**S3) Exp. 1 – Methods – Post experimental inquiry and questionnaires**

After the experiment, participants were interviewed again with respect to suspicions about the purpose of the study, their burden due to the duration of the study, and the use of specific strategies when observing the starfield and answering the questions (e.g. counting). Afterwards, participants were asked to fill out three questionnaires in paper form, the ‘Beck Depression Inventory’(BDI^1^), the ‘Autism Quotient’ (AQ^2^) and the ‘Boredom Proneness Scale’ (BPS^3^). Questionnaire data will be analyzed quantitatively in the future and served exploratory purposes.

**S4) Exp. 1 – Results – Perceptual validity checks**

The low number of trials including velocity and density ratings per subject did not allow fitting random intercepts for participants. Instead, average differences in velocity and density ratings between low and high velocity and density trials were calculated and their upward deviation from zero was tested in one-sided one-sample t-tests. Both, higher VELOCITY t(19) = 9.37, p < .001; Low velocity: M = 21.95, SD = 12.89; High velocity: M = 63.25, SD = 16.78) and higher DENSITY (t(19) = 4.02, p < .001; Low density: M = 48.25, SD = 22.93; High density: M = 70.95, SD = 19.68) significantly increased their respective measurements.

**S5) Exp. 1 – Results – Effects of participant variables**

We analyzed whether the participants reports of their experience of how fast their life passes in general was related to their average DoTJ or PoTJ. We computed the means either for DoTJ or PoTJ (depending on the participants experimental group) and assessed the correlation to their experience of passage of life time. We did not find a significant correlation for mean DoTJs (r = -0.15, p = .175). However, a weak but significant correlation could be observed between mean PoTJs and one´s own experience of passage of life time (r = .23, p = .023).

Similarly, we tested whether the participants’ demographics were related to their average DoTJs or PoTJs. Therefore, corresponding to the procedure for between subject analysis of the effects of starfield velocity and density, we tested whether including age, gender or years of education would improve the fits of linear models in likelihood ratio tests. However, we did not find any significant relationships for DoTJs (age: F(1,80) = 0.13, p = .714; gender: F(1,80) = 1.31, p = .256; education: F(1,80) = 2.18, p = .144) or PoTJs (age: F(1,95) = 0.72, p = .398; gender: F(1,94) = 2.12, p = .149; education: F(1,95) = 0.17, p = .682). Note that in the analysis of the effect of gender it was not possible to analyse the effect of the ‘diverse’ gender due to the small sample (n=1).

**S6) Exp. 2 – Methods – Data-quality assessment**

In 28 participants the experiment was not correctly conducted due to technical issues in the assignment of the experimental condition, 16 participants reported problems with the playback of the videos (e.g. due to internet connection issues), 2 participants reported disturbances or interruptions during participation (e.g. phone ringing or someone entering the room) that exceeded the length of one trial; in 2 persons, the total duration suggested that they had interrupted participation and only continued after a longer break; in 2 participants the timestamps of video playbacks suggested some technical issues with the presentation (e.g. due to internet connection issues); 2 participants did not pass the initial color detection test (see section Online Specific Procedures); 33 participants did not reach the required average accuracy score (>=.8) in color detection, suggesting reduced attention during the experiment.

**S7) Exp. 2 – Methods – Stimuli**

For Exp. 2, a rudimentary sphere-effect was added to the display of stars by applying a brightness gradient (increased brightness in the center, decreased in the periphery). In addition, stimuli were saved as video files for the presentation in the online experiment. Video files had a resolution of 1280 * 720 pixel and 24 fps.

Compared to Exp. 1, due to the lower resolution of the video and potentially smaller display devices, fewer stars were depicted in Exp. 2 in both the low (188 stars instead of 750) and the high density condition (750 stars instead of 3000).

As in Exp. 1, changes in the starfield’s color ratio did not happen abruptly but gradually with each star that was assigned a new color when reaching the front and reappearing in the back (see section Stimuli in Exp. 1 – Methods). In order to secure similar durations of color changes during different velocities, the probability of each star changing its color was adjusted to its speed. Faster stars had a smaller probability of changing the color when reappearing. Additionally, we ensured that also after the last change there was a minimal time of 1.5 seconds with a predominance of at least 75% for one color.

**S8) Exp. 2 – Methods – Procedures specific to the online format**

Participants who demonstrated their interest received a link in their Prolific (www.prolific.co) profile, forwarding them to the platform Unipark (www.unipark.de) where the study was implemented. Here, participants received detailed information in written format about the study and gave their consent.

Afterwards, participants were instructed to switch their browser to a full screen display and to keep these settings until further notice at the end of the experiment. Participants then filled out a short demographic questionnaire. As a test of their display settings and their ability to perceive the stars correctly, participants were then presented with a still of the starfield and were instructed to report the number of stars of each color (correct numbers ranging from 6 – 9). Only participants who reported the correct number (including an error margin of ± 1) were later included in the analysis.

On the next page, participants were then instructed to stretch out their arm in front of them with a raised thumb and to check, whether the tip of their thumb would cover a black circle, displayed on the screen. In case it did not, participants were instructed to adjust the zoom factor in their browser until the tip of their thumb would barely cover the circle. Due to the fairly high stability of the ratio between arm length and thumb width between humans^4^, this procedure ensured that the size of the starfield stimulus in the visual field would be similar between participants. The adjusted zoom factor was then maintained throughout the experiment and was applied to the starfield stimulus presentation.

After these adjustments, participants received task specific instructions and conducted the trials (see ‘Task’ section below).

The experiment was followed by a few additional questions. First, participants were asked how fast their live passes in general, again via the same scale used for PoTJs in the main experiment. Afterwards, participants were asked to i) provide some basic information about the screen on which they had conducted the experiment by describing it in general as well as estimating the size of its diagonal, ii) report whether they had noticed any problem with the display of the stimuli or the experiment in general and to describe the issues as detailed as possible, and iii) to describe any events that occurred during their participation that could potentially have disturbed or interrupted them.

**References**

1. Beck, A. T., Steer, R. A. & Hautzinger, M. *Beck-Depressions-Inventar (BDI): Testhandbuch*. (Huber, 2001).

2. Baron-Cohen, S., Wheelwright, S., Skinner, R., Martin, J. & Clubley, E. The autism-spectrum quotient (AQ): Evidence from asperger syndrome/high-functioning autism, malesand females, scientists and mathematicians. *J. Autism Dev. Disord.* **31**, 5–17 (2001).

3. Farmer, R. & Sundberg, N. D. Boredom proneness--the development and correlates of a new scale. *J. Pers. Assess.* **50**, 4–17 (1986).

4. O’Shea, R. P. Thumb’s Rule Tested: Visual Angle of Thumb’s Width is about 2 Deg. *Perception* **20**, 415–418 (1991).
